# Supplementary material for: The “Ifs” and “Hows” of the Role of Music on the Implementation of Emotional Regulation Strategies
Source: Behav Sci (Basel). 2022 Jun 20;12(6):199. doi: 10.3390/bs12060199 (PMC9219814; doi:10.3390/bs12060199)
Supplement: Supplementary file 1 [file behavsci-12-00199-s001.zip › S3. Model comparisons.pdf]

Comparison of models engaging executive functions (lowest AIC)

analysis1: RE ~ Retype * condSTRA * condMU + (1 | ID)
analysis2: RE ~ Retype * condSTRA * condMU * MD + (1 | ID)
analysis4: RE ~ Retype * condSTRA * condMU * NA.P + (1 | ID)
analysis5: RE ~ Retype * condSTRA * condMU * NA.N + (1 | ID)
analysis6: RE ~ Retype * condSTRA * condMU * A.N + (1 | ID)
analysis7: RE ~ Retype * condSTRA * condMU * A.P + (1 | ID)
analysis104: RE ~ Retype * condSTRA * condMU * StroopAbs + (1 | ID)
analysis23: RE ~ Retype * condSTRA * condMU * MD * NA.N + (1 | ID)
analysis101: RE ~ Retype * condSTRA * condMU * StroopAbs * NA.N + (1 | ID)
analysis102: RE ~ Retype * condSTRA * condMU * StroopAbs * MD + (1 | ID)

	Df	**AIC**	BIC	logLik	deviance	Chisq	Chi Df	Pr(>Chisq)	
analysis1	10	167.53	193.18	-73.767	147.53				
analysis2	18	170.79	216.94	-67.393	134.79	12.7468	8	0.1209	
analysis4	18	181.07	227.23	-72.537	145.07	0.0000	0	1.0000	
analysis5	18	164.13	210.28	-64.064	128.13	16.9468	0	<2e-16	***
analysis6	18	175.65	221.81	-69.827	139.65	0.0000	0	1.0000	
analysis7	18	168.15	214.31	-66.077	132.15	7.5006	0	<2e-16	***
analysis104	18	170.24	216.40	-67.121	134.24	0.0000	0	1.0000	
**analysis23**	**34**	**161.97**	**249.16**	**-46.987**	**93.973**	**34.154**	**16**	**0.005179**	
analysis101	34	167.79	254.98	-49.897	99.793	0	0	1	
analysis102	34	179.20	266.38	-55.598	111.196	0	0	1	
