# Supplementary material for: The “Ifs” and “Hows” of the Role of Music on the Implementation of Emotional Regulation Strategies
Source: Behav Sci (Basel). 2022 Jun 20;12(6):199. doi: 10.3390/bs12060199 (PMC9219814; doi:10.3390/bs12060199)
Supplement: Supplementary file 1 [file behavsci-12-00199-s001.zip › S2.Executive Functioning Tasks.pdf]

Results

Correlation

Pearson's Correlations

Variable		StroopAbs	NA|P	NA|N	A|P	A|N	MD
1. StroopAbs	Pearson's r	—					
	p-value	—					
2. NA|P	Pearson's r	0.062	—				
	p-value	0.675	—				
3. NA|N	Pearson's r	−0.198	0.221	—			
	p-value	0.178	0.131	—			
4. A|P	Pearson's r	−0.117	0.080	−0.186	—		
	p-value	0.428	0.591	0.206	—		
5. A|N	Pearson's r	0.259	−0.146	−0.167	0.022	—	
	p-value	0.076	0.322	0.256	0.885	—	
6. MD	Pearson's r	0.076	−0.087	−0.079	0.115	−0.158	—
	p-value	0.609	0.556	0.593	0.438	0.282	—

* p < .05, ** p < .01, *** p < .001

Exploratory Factor Analysis

Chi-squared Test

	Value	df	p
Model	0.000	-3	

Warning: Degrees of freedom below 0, model is unidentified.

Factor Loadings

	Factor 1	Factor 2	Factor 3	Factor 4	Uniqueness
StroopAbs	1.084				0.200
NA|P		1.015			0.282
A|P			0.920		0.340
MD				0.608	0.646
NA|N					0.776
A|N					0.706

Note. Applied rotation method is promax.

Factor Characteristics

	Unrotated solution			Rotated solution		
	SumSq. Loadings	Proportion var.	Cumulative	SumSq. Loadings	Proportion var.	Cumulative
Factor 1	1.021	0.170	0.170	1.006	0.168	0.168
Factor 2	0.829	0.138	0.308	0.856	0.143	0.310
Factor 3	0.751	0.125	0.433	0.727	0.121	0.431
Factor 4	0.449	0.075	0.508	0.461	0.077	0.508

Factor Correlations

	Factor 1	Factor 2	Factor 3	Factor 4
Factor 1	1.000	−0.517	0.413	−0.273
Factor 2	−0.517	1.000	−0.437	0.190
Factor 3	0.413	−0.437	1.000	−0.047
Factor 4	−0.273	0.190	−0.047	1.000
